# SamQL: a structured query language and filtering tool for the SAM/BAM file format

**DOI:** 10.1186/s12859-021-04390-3

**Published:** 2021-10-02

**Authors:** Christopher T. Lee, Manolis Maragkakis

**Affiliations:** Laboratory of Genetics and Genomics, National Institute on Aging, Intramural Research Program, National Institutes of Health, Baltimore, MD 21224 USA

**Keywords:** Genomics, SQL, SAM, BAM, Big data

## Abstract

**Background:**

The Sequence Alignment/Map Format Specification (SAM) is one of the most widely adopted file formats in bioinformatics and many researchers use it daily. Several tools, including most high-throughput sequencing read aligners, use it as their primary output and many more tools have been developed to process it. However, despite its flexibility, SAM encoded files can often be difficult to query and understand even for experienced bioinformaticians. As genomic data are rapidly growing, structured, and efficient queries on data that are encoded in SAM/BAM files are becoming increasingly important. Existing tools are very limited in their query capabilities or are not efficient. Critically, new tools that address these shortcomings, should not be able to support existing large datasets but should also do so without requiring massive data transformations and file infrastructure reorganizations.

**Results:**

Here we introduce SamQL, an SQL-like query language for the SAM format with intuitive syntax that supports complex and efficient queries on top of SAM/BAM files and that can replace commonly used Bash one-liners employed by many bioinformaticians. SamQL has high expressive power with no upper limit on query size and when parallelized, outperforms other substantially less expressive software.

**Conclusions:**

SamQL is a complete query language that we envision as a step to a structured database engine for genomics. SamQL is written in Go, and is freely available as standalone program and as an open-source library under an MIT license, https://github.com/maragkakislab/samql/.

**Supplementary Information:**

The online version contains supplementary material available at 10.1186/s12859-021-04390-3.

## Background

The advent of high-throughput sequencing has created an unprecedented availability of genomic data, highlighting the need for efficient storage and processing of large datasets. To address these requirements the Sequence Alignment Map (SAM) and its binary equivalent (BAM) file formats were developed [1]. These file formats together with the later developed CRAM [2], have been adopted by many bioinformatics software, including almost all alignment programs [3–6]. Each record in the SAM format has several descriptive fields including alignment coordinates, sequence information, sequence and mapping quality and others.

Along with these file formats, SAMtools and other specialized programs [7–10] were developed to enable processing and access to the encoded data. In addition, specialized data formats such as ArrowSAM [11] have started to be introduced, focusing on columnar data representations of SAM data that can allow faster data retrieval, sometimes 15 × or 2.4 × greater than existing implementations of Picard [9] and Sambamba [8]. Despite these developments, complex queries on genomics data remain a bottleneck and are substantially difficult to perform due to the lack of a uniform query language. The web is flooded with related questions from investigators that usually end up using custom Bash commands. Additionally, while SAMtools and other programs have a wide variety of functions and options, they remain unintuitive and require the user to often refer to the documentation leading to coding time increase and possibly inefficient or, more importantly, error-prone software.

These limitations are now becoming even more apparent due to the rapid increase in the size of genomic data [12] that is introducing new challenges regarding access, security and data management [13]. Existing tools are unable to address these challenges due to performance or query capabilities shortcomings. To address these limitations, a new structured database engine and a supporting expressive query language that can allow organized genomic data access is required.

Here we introduce SamQL, a command line tool and library, that allows for SQL-like queries on top of the SAM/BAM format. SamQL has intuitive syntax allowing complex queries and takes advantage of parallelizable handling of BAM files. SamQL works on top of SAM/BAM formats to avoid data transformations to other formats and thus simplifies adoption. However, the engine is extendable to support future, more performant formats such as ArrowSAM as they are adopted by the community. SamQL aims to eventually enable the development of a genomics database on top of SAM/BAM or other more performant data structures, enabling complex queries for genomic datasets.

## Implementation

SamQL aims to provide a user-friendly syntax with high expressive power to support a parallelizable, database for genomics. SamQL was developed in the Go programming language. Internally, SamQL uses two robust and flexible bioinformatics libraries biogo [14] and hts [15] that provide a clean interface to the common bioinformatics file formats. SamQL comprises a complete lexer that performs lexical analysis and a parser, that together analyze the syntax and process the provided query.

**Fig. 1:**
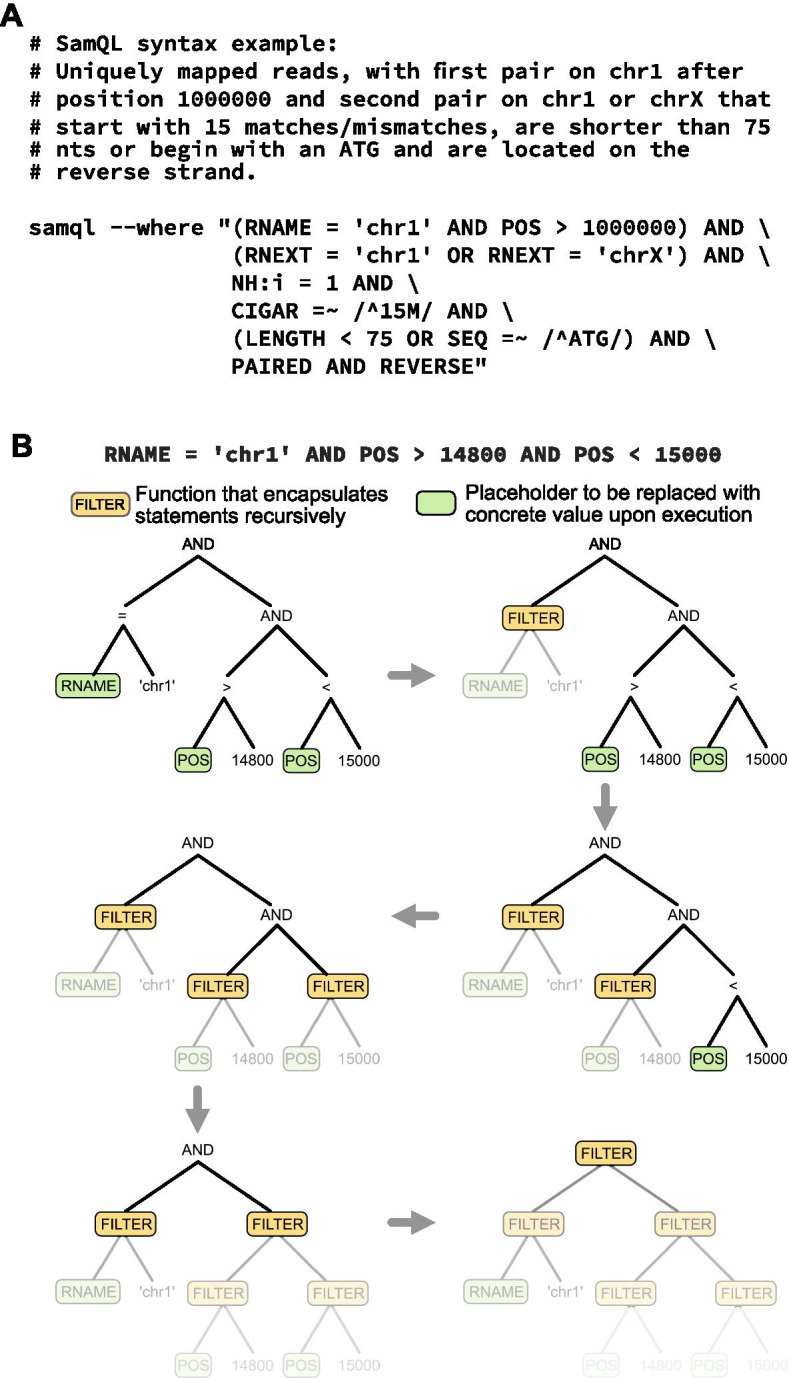
SamQL offers an intuitive and structured query language for SAM/BAM files. **A** Example of a complex SamQL query. SamQL can support searching on any fields of interest, including optional fields and flags, with intuitive syntax similar to standard SQL. **B** Schematic of the execution algorithm and the abstract syntax tree constructed from the query. A FILTER function returns a single boolean value that encapsulates all filters below. The whole tree is progressively replaced by a single FILTER function that is used for record filtering

We designed SamQL queries to look similar to SQL queries that are widely adopted in computer science, to make the system intuitive to use and to substantially lower the initial learning curve. To support SAM-specific data extraction, SamQL recognizes SAM fields by their corresponding names defined in the SAM specification (i.e. QNAME, FLAG, RNAME, POS, MAPQ, CIGAR, RNEXT, PNEXT, TLEN, SEQ, QUAL) and assigns them as language keywords. These keywords are dynamically replaced by the actual concrete values upon code execution. Additional keywords have been added to support the SAM encoded flag field significantly simplifying access to this information (e.g. PAIRED, SECONDARY, etc.). The SamQL model is flexible and additional keywords can be added to support future requirements. For example, the LENGTH keyword has been added to correspond to the alignment length and is automatically evaluated upon execution. Figure 1A shows an example of the SamQL syntax and highlights the flexibility of the query system. Importantly, any query in SamQL is evaluated once at the beginning of the search, making the model lightweight and reducing computation time. To support this, SamQL builds an abstract syntax tree (AST) corresponding to the query. The AST is then parsed, depth-first, to progressively build a function closure that encapsulates the whole query (Fig. 1B). The closure contains the entire filtering criteria, can accept a SAM record for filtering, and returns a boolean value indicating whether the record passes all criteria.

## Results

Our primary aim building SamQL was flexibility and high expressivity for complex queries, similar to classic SQL. Table 1 compares the expressivity of SamQL for a relatively complex query against other widely used tools such as SAMtools, Sambamba, and naive Bash. SamQL maintains consistency on complex queries involving coordinates. However, Sambamba and samtools separate the range query from the main filtering criteria. This leads to less consistent syntax as highlighted in the example in Table 1.

**Table 1:** Comparison of syntax for a reasonably complex query to keep reads on chromosome 2, mapping at position greater than 1,000,000, on the reverse strand, with mapping quality higher than 30

Tool	Syntax
samql	RNAME = "chr2" AND POS > 1000000 AND REVERSE AND MAPQ > 30
samtools	chr2:1000000 -f 16 -q 30
sambamba	'reverse_strand and mapping_quality > 30' chr2:1000000
bash	… | awk -F '\t' '{if ($3 = = "chr2" && $4 > 1000000 && and($2,16) && $5 > 30) print}'

Sambamba has similar expressivity to SamQL but like samtools, region queries are separated from the main filters, resulting in less intuitive syntax. We have excluded bamtools because it requires a JSON file for advanced queries

SamQL is a complete query language with a lexer and parser specifically designed for genomic data in the SAM/BAM format. This enables SamQL to output more informative messages for potential syntax errors. Table 2 shows a specific example for a simple syntax error involving a misplaced closing parenthesis. SamQL returns information about the specific type of the error and the position at which the error occurred. In contrast, neither samtools nor sambamba provide such information.

**Table 2:** Comparison of the error messages of the different tools for a syntax error involving a misplaced parenthesis

Tool	Syntax	Error message
samql	(POS > 1 AND) (RNAME = "chr1")	Filter creation from where clause failed: found), expected identifier, string, number, bool at line 1, char 12
samtools	(pos > 1 &) (rname = = "chr1")	Couldn't process filter expression
sambamba	(position > 1 and) (ref_id = = 0)	Parsing error near line 1: expected prefix operator

Besides expressivity we also wished to test the computational performance of SamQL. We compared SamQL against other widely used tools; SAMtools, Sambamba, BamTools and naive Bash, essentially approaching daily used workflows by bioinformaticians. We tested on three different queries, each one with varying levels of syntax complexity and computational requirements. We repeated each query 10 times on varying input sizes to validate the variation and accuracy of our measurements. All comparisons were run on an Intel Xeon, 48 core, 384 Gb memory compute server. We used a publicly available BAM file with 3,363,576 records. To evaluate scalability in terms of input size, we randomly sampled the input BAM file into sizes of 10% increments and performed measurements on all subsets individually. To evaluate the query performance and decouple it from Input/Output (IO) we measured the execution time both when printing to an output file but also just counting the filtered reads. We also tested SamQL by limiting it to 2 execution threads or leaving it unbound to automatically scale to the available resources.

**Fig. 2:**
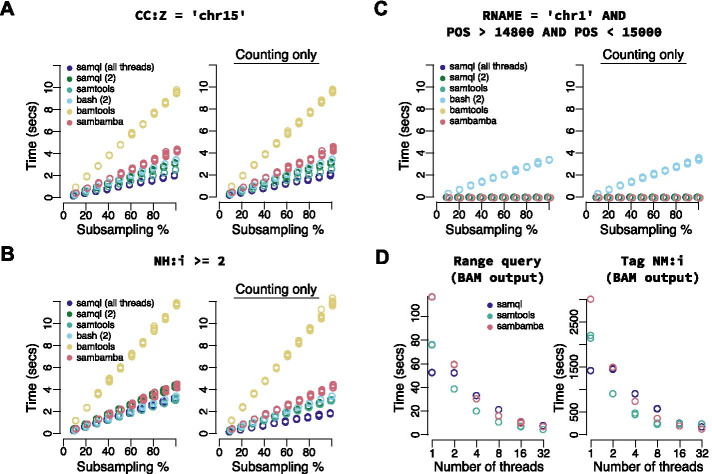
SamQL performance. **A**–**C** From left to right, the plots correspond to runtime for printing and counting SAM entries on increasing subsets of the input data for different queries. The used queries are: A string query on tag “CC:Z” (**A**); a numeric query on tag NH:i (**B**); A range query (**C**). The corresponding SamQL query is shown at the top. **D** Parallelization performance for BAM output for a range query (left) and the NH:i tag (right) on a large BAM dataset of approximately 900 million reads. For NH:i only 10% of the file was processed to keep the execution times reasonable. SamQL is inherently concurrent and cannot be limited to less than 2 threads which is why performance is equivalent at 1 and 2 threads. Colors correspond to SamQL using all threads (dark blue), SamQL bound to 2 cores (green), SAMtools (light green), naive Bash/Awk (cyan), BamTools (yellow) and Sambamba (red). The raw data for the plots can be found in Additional file 1: Table S1.

As a first test, we decided to compare performance on filtering against a SAM tag that uses string matching and is supported by all methods, CC:Z. Filtering on optional tags forces all methods to read the entire SAM record thus decoupling IO optimizations that depend on skipping optional SAM fields. We find that SamQL performs on par with the other methods even when bound to use just two threads, one for IO and one for compute (Fig. 2A). As a next test we wished to filter on the NH:i tag that involves numerical comparisons. This is an intuitive and straightforward query change in SamQL (Fig. 2B, top) and Sambamba. However, for SAMtools we had to use the newly added -e option instead of the -d to support numeric comparisons. As expected, the naive Bash implementation becomes substantially more complex also raising the possibility for coding mistakes. Again, we find that SamQL execution time is on par or faster than other methods and that execution time again increases linearly with time (Fig. 2B). Overall, we find that most methods perform similarly, except BamTools that is substantially slower than others. We also notice that SamQL multithreading does speed up execution time but only modestly. Importantly, we find that execution time increases linearly with the input size which is a key feature that allows SamQL to be used for large datasets.

Next, we evaluated the SamQL performance for a reasonably complex range query. Range queries on genomic or transcriptomic coordinates are among the most used query types in bioinformatics analyses. Therefore, BAM files are usually indexed to achieve fast retrieval of alignments that overlap a given region [16]. SamQL can execute range queries on indexed and not indexed BAM or SAM files, albeit much faster when indexing exists. Our data show that SamQL performs on par with the other software and orders of magnitude faster than a Bash approach (Fig. 2C).

The file that we used for evaluating performance although it serves for micro-benchmarks and we have seen linear scalability with size, it does not necessarily simulate real-world usage that might involve tens or hundreds million reads. Therefore, we performed similar benchmarks for a publicly available file of much larger size, approximately 900 million reads. Our results again show that range queries for all tools are executed much faster than naive Bash (Additional file 1: Fig. S1A) and comparable with each other. To gain more insight as to the scalability of the tools to very large datasets and to measure parallelization performance more accurately, we used this larger dataset to run all tools at different parallelization settings, progressively increasing the thread usage. We scaled the tools from 1 to 32 threads to monitor how efficiently the tools use the available resources. We tested a range query on the full file and the filter on the NM:i tag on a smaller, 10%, subset of the file due to very high runtimes for all tools. Initially we focused on SAM output. Interestingly we found that SamQL does not scale as well as the other tools reaching a performance plateau at approximately 4 threads (Additional file 1: Fig. S1B). However, we find that when BAM output is requested the scalability of the tool improves similarly to SAMtools and Sambamba. This indicates that BAM parallelization at the output provides substantial benefits. As expected, we find that the runtime of all tools reduces but with diminishing returns at they get close to 32 threads. Overall, all our tests indicate that SamQL offers high expressivity for complex queries while also achieving high performance and being able to utilize and take advantage of parallel computing.

## Conclusions

Compared to existing tools SamQL offers intuitive and simpler syntax and is closer to traditional SQL that most scientists are familiar with. It can therefore be easier to adopt and use by bioinformaticians and non-bioinformaticians. SamQL can replace most one-liners used by bioinformaticians, thus helping to reduce errors. Also, SamQL can support complex queries that are straightforward to express and are orders of magnitude faster than naive Bash implementations when range queries are involved. Finally, it is the only one from the tested tools, that can seamlessly query more than one files. While SamQL can utilize and benefit from multicore systems, we find that performance improvement plateaus at approximately 4 threads when SAM output is requested, indicating that there is room for more optimization. Interestingly, no such plateau is observed for BAM output which benefits from parallelization of the output layer. Potential future optimizations can include parallelization of subexpressions similar to other database engines or extending the underlying data storage to a columnar format such as ArrowSAM [11] which has been shown to increase processing speed substantially. The future goal for SamQL would be to act as a query language for the development of a complete, genomic database, that can be adopted the same way that SQL was adopted for relational databases in computer science. We envision a database running on top of existing SAM/BAM files where investigators would be able to easily search through every file source for reads of interest.

## Supplementary Information

**Additional file 1. Figure S1**. (**A**) From left to right, the plots correspond to runtime for printing and counting SAM entries for a range query shown at the top on increasing subsets of a large input dataset of approximately 900 million reads. (**B**) Parallelization performance for SAM output for a range query on the same file as in (**A**)

**Additional file 2. Table S1**. Raw runtime data of query methods used to create Fig. 2.

## Data Availability

The datasets analyzed during the current study are available in http://hgdownload.cse.ucsc.edu/goldenpath/hg19/encodeDCC/wgEncodeCaltechRnaSeq/wgEncodeCaltechRnaSeqGm12878R1x75dSplicesRep1V2.bam and ftp://ftp-trace.ncbi.nlm.nih.gov/ReferenceSamples/giab/data/AshkenazimTrio/HG002_NA24385_son/NIST_Illumina_2x250bps/novoalign_bams/HG002.GRCh38.2x250.bam.

## Abbreviations

AST: Abstract syntax tree
BAM: Binary SAM
IO: Input/output
SAM: Sequence alignment/map format specification
SQL: Structured query language
