# Supplementary figures and images for: SamQL: a structured query language and filtering tool for the SAM/BAM file format
Source: BMC Bioinformatics. 2021 Oct 2;22:474. doi: 10.1186/s12859-021-04390-3 (PMC8487582; doi:10.1186/s12859-021-04390-3)

**A**

RNAME = 'chr20' AND POS > 1 AND POS < 10000000

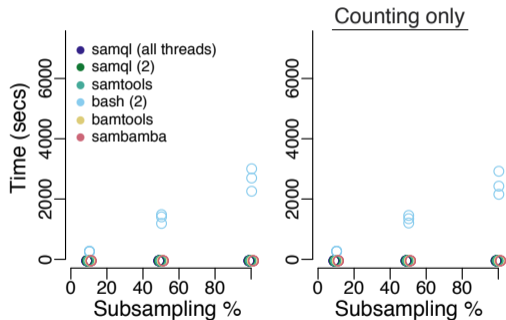**B**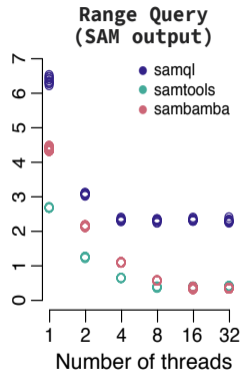

Supplement: Supplementary file 1 — Additional file 1. Figure S1. (A) From left to right, the plots correspond to runtime for printing and counting SAM entries for a range query shown at the top on increasing subsets of a large input dataset of approximately 900 million reads. (B) Parallelization performance for SAM output for a range query on the same file as in (A) [file 12859_2021_4390_MOESM1_ESM.pdf]
